# Remote Monitoring: How to Maximize Efficiency through Appropriate Organization in a Device Clinic

**DOI:** 10.3390/jcdd11090270

**Published:** 2024-08-30

**Authors:** Massimiliano Maines, Giancarlo Tomasi, Luisa Poian, Marzia Simoncelli, Debora Zeni, Monica Santini, Maurizio Del Greco

**Affiliations:** Department of Cardiology, Santa Maria del Carmine Hospital—APSS Trento, Corso Verona 4, 38068 Rovereto, TN, Italy; giancarlo.tomasi@apss.tn.it (G.T.); luisa.poian@apss.tn.it (L.P.); marzia.simoncelli@apss.tn.it (M.S.); maurizio.delgreco@apss.tn.it (M.D.G.)

**Keywords:** devices remote monitoring, organization model, sustainability

## Abstract

Introduction: Remote device monitoring is indicated under class I A standard of care according to the latest HRS/EHRA/APHRS/LAHRS Expert Consensus Statement on Practical Management of the Remote Device Clinic. Despite this strong endorsement and the supporting data, the adoption of remote monitoring practices remains lower than expected. One cause of the underutilization of telemonitoring devices is work overload. Thus, a crucial point for improving the adoption of remote monitoring systems is ensuring their sustainability. Materials and Method: After analyzing the resources necessary to manage a device telemonitoring clinic, we initiated a process to reduce redundant transmissions: 1. eliminated scheduled loop recorder transmissions, retaining only alert transmissions; 2. reduced the frequency of the scheduled transmissions of pacemakers from four to one per year and the scheduled transmissions for defibrillators from four to two per year; and 3. optimized and customized the programming of device alerts with two primary interventions. Results: These strategies allowed us to significantly reduce the number of transmissions/patient/year from 7.3 to 4.7. The first change was made in January 2020, which eliminated scheduled transmissions for loop recorders, reduced transmissions per patient from 14 to 10.4 for loop recorders, and decreased global transmissions per patient from 7.6 to 6.5. The subsequent adjustment in January 2021, which reduced the scheduled transmissions of pacemakers and defibrillators, further lowered transmissions per patient from 6.5 to 5.2 for pacemakers and from 4.7 to 3.1 for defibrillators. Additionally, enhanced attention to device reprogramming starting in January 2022 resulted in a further reduction in transmissions per patient from 5 to 4.7. Conclusion: Carrying out some simple changes in the number of scheduled transmissions and optimizing the programming of the devices made it possible to reduce the number of transmissions and make the remote monitoring of the devices more sustainable

## 1. Introduction

A series of studies have demonstrated the advantages of remote monitoring in several key areas: reducing response times to clinical problems with devices [1,2]; improving the survival rates of patients with remotely monitored devices [3,4]; and decreasing hospitalizations [5], urgent visits [6], and outpatient visits [7]. Remote device monitoring can also be a valuable resource in the daily management of patients with heart failure [8]. Due to the substantial evidence from these studies, remote device monitoring has been classified under class IA standard of care for these patients according to the latest HRS/EHRA/APHRS/LAHRS Expert Consensus Statement on Practical Management of the Remote Device Clinic [9]. Despite this strong endorsement and the supporting data, the adoption of remote monitoring practices remains lower than expected. According to the AIAC Survey published in 2021 [10], the second leading cause of the underutilization of device telemonitoring in Italy is work overload, with the primary cause being the lack of recognition for performance in many contexts. Thus, a crucial point for improving the adoption of remote monitoring systems is ensuring their sustainability. This involves reducing non-significant or redundant transmissions, thereby allowing healthcare providers to focus more time on patients with actual clinical issues.

## 2. Materials and Methods

Our telecardiology clinic in Rovereto (TN, Italy) is staffed with three nurses who have undergone specialized training within our hospital. On average, two nurses are always on duty. They deliver the remote monitoring system to patients (before discharge, if possible, in the presence of a caregiver or a patient’s family member), perform remote monitoring of devices, and respond to patient phone calls. Two nurses are also certified for the implantation of loop recorders. On pre-established days, they perform these implantations, supported by a doctor who provides informed consent for the procedure and drafts the outpatient report. Subsequently, they provide remote monitoring, ensuring a comprehensive patient care process.

We have established procedures to manage various device alerts [11,12], with a reference doctor available every afternoon for two hours to address significant clinical issues.

After analyzing the resources necessary to manage a device telemonitoring clinic, we initiated a process to reduce redundant transmissions:

1. In January 2020, we eliminated scheduled loop recorder transmissions, retaining only alert transmissions [13].
2. In January 2021, following the publication of our analysis on the volume of transmissions generated by scheduled interrogations [14], we reduced the frequency of the scheduled transmissions of pacemakers from four to one per year and the scheduled transmissions for defibrillators from four to two per year.
3. Since January 2022, we have been optimizing and personalizing the programming of device alerts with two primary interventions:

Reprogramming loop recorders that generated many false alerts.

Extending the cut-off for the detection of atrial high-rate episode burden to 24 h for patients already on anticoagulant therapy (while keeping the alert active for atrial fibrillation with high ventricular response).

### Statistical Analysis

Transmission rates were computed for each calendar year in the study population and represented as events per patient year by calculating the ratio between the event counts and their respective follow-up durations. A *p* value of <0.05 was considered significant for all tests. All statistical analyses were performed by means of R: a language and environment for statistical computing (R Foundation for Statistical Computing, Vienna, Austria).

## 3. Results

The number of patients followed at the center and the volume of procedures carried out from 2018 to 2023 are reported in Table 1. The device reprogramming and optimization policy has allowed us to reduce the number of transmissions per patient over time, despite the increase in the number of devices implanted and controlled remotely. The change made in January 2020, which eliminated scheduled transmissions for loop recorders, reduced transmissions per patient from 14 to 10.4 for loop recorders and decreased global transmissions per patient from 7.6 to 6.5. The subsequent adjustment in January 2021, which reduced the scheduled transmissions of pacemakers and defibrillators, further lowered transmissions per patient from 6.5 to 5.2 for pacemakers and from 4.7 to 3.1 for defibrillators. Additionally, enhanced attention to device reprogramming starting in January 2022 resulted in a further reduction in transmissions per patient from 5 to 4.7.

## 4. Discussion

The heart of telecardiology lies not only in the technology that allows the remote monitoring of devices but, more importantly, in the organizational model. In many telemedicine studies, telecardiology has yielded variable results. However, as demonstrated by the IN-TIME study [4], telecardiology significantly impacts important endpoints when the organizational model ensures prompt responses to alerts. The successful model, as documented in the literature, involves dedicated clinics with trained nurses and technicians who monitor patients with devices. These healthcare professionals are responsible for remote monitoring, reviewing transmissions, and addressing patient questions about the devices, with support from doctors when necessary. Educating patients about the benefits and limitations of remote monitoring is essential for their engagement. Providing clear instructions on using RM equipment and setting expectations can reduce anxiety and improve compliance. Continuous education and certification programs for nurses and technicians are vital to maintaining high standards of care. Device manufacturing companies can play a fundamental role in this by offering training and updates [9]. Additionally, manufacturers are crucial in promptly informing healthcare providers about device recalls and managing technical problems. Another critical aspect of remote monitoring is ensuring reliable connectivity, which is essential for providing quality service. Each center should have procedures in place to verify and guarantee patient connectivity. The organization must have clear protocols for data review and patient management. These protocols should outline the responsibilities of each team member, define criteria for escalating care, and establish timelines for reviewing and responding to alerts [9]. Informed consent is also crucial. Patients must be informed about what remote monitoring can provide, the center’s response times based on available resources, and the procedures in place. This ensures that patients understand and consent to the monitoring process.

In 2016, at Rovereto Cardiology, we followed approximately 1800 device patients in person and conducted around 5000 visits and device checks per year, resulting in waiting times of eight months for a visit. At that time, we performed around 300–350 device implantations per year, and this would have made the working model unsustainable, with the need for additional medical staff to manage the patient follow-up. The 2015 HRS consensus document included a Class I recommendation for remote monitoring for patients with a recalled device and classified remote monitoring as equivalent to in-person monitoring. Therefore, in 2017, we decided to provide all our patients with remote control/monitoring for their devices. With the technological change, it was also necessary to rethink our organization. Consequently, we established a nursing clinic for the remote monitoring of devices, with a dedicated nurse and a doctor providing support for two hours a day. In our organization, all pacemaker operations were transitioned from in-office visits to remote control, with in-person visits as needed. ICDs maintained 1 in-person check-up per year in addition to remote monitoring, while biventricular devices were monitored remotely with 1–2 check-ups per year in our heart failure clinic. This approach reduced our waiting lists from 8 months to 18 days by the end of 2018, saving our patients 200 km/year per patient. In terms of clinic staff, we calculated that one dedicated nurse and 0.14 of a doctor’s time were needed to monitor 1000 patients with our organizational model. However, over time, the number of implantations and, consequently, the number of patients monitored remotely increased. As of December 2018, the average number of transmissions per patient was 11.7. We began thinking about optimizing our organizational model, believing it was essential for the success of telecardiology to make the model sustainable and manageable with a limited number of staff.

Ways to optimize information flows include the following:

1. Maintaining clinically meaningful transmissions with events and reducing redundant ones: Indeed, our work published in 2021 demonstrated that alert transmissions generate a greater need for medical supervision and additional in-person evaluations compared to scheduled ones. Therefore, in our model, we reduced scheduled transmissions (one/year for pacemakers, two/year for defibrillators and biventricular devices, and no scheduled transmissions for loop recorders) [14].
2. Correctly programming the devices and reprogramming those that transmit frequently [13] or optimizing the alerts that are no longer clinically meaningful: for example, if the patient goes into permanent atrial fibrillation, the atrial fibrillation alert can be turned off.

Another proposal to optimize the organization could be to create telecardiology centers for large areas that manage patients from multiple hospitals, referring them to the reference center in case of problems. This model requires the implementation of the electronic health record visible to the centers involved and would allow resources to be optimized even if the overall care of the patient by the implanting center is lost, which is also the basis of the relationship established with the patients.

## 5. Limitations

In our study, we evaluated the impact of certain organizational changes, such as reducing scheduled transmissions and optimizing device programming, on workload. However, we did not assess the potential impact on clinical outcomes. Previous studies have suggested that an alert-based management approach is not inferior to structured intermittent device follow-up in terms of safety. This approach has also been associated with the almost immediate detection of actionable events, improved patient retention, enhanced follow-up, and better quality of life [15]. While it is plausible that increased efficiency could lead to improved patient care, further studies are needed to substantiate this.

## 6. Conclusions

Some simple changes in the number of scheduled transmissions and optimizing the programming of the devices made it possible to reduce the number of redundant transmissions and make the remote monitoring of the devices more sustainable.

## Figures and Tables

**Table 1:** Number of patients followed at the center and the volume of procedures carried out from 2018 to 2023.

	2018	2019	2020	2021	2022	2023
Patients with monitored devices	1887	2029	2309	2378	2625	2741
Pacemaker	1078	1120	1274	1337	1485	1571
Defibrillators	402	406	423	393	402	418
ILR	407	503	612	648	738	752
Transmissions	13,859	15,414	14,954	12,453	13,084	12,775
Pacemaker	5714	5996	5991	4107	4823	4837
Defibrillators	2416	2376	2554	1672	1693	1546
ILR	5729	7042	6409	6674	6568	6392
Nurses	1	2	2	2	2	2
Transmissions/patient year #	7.3 (7.2–7.5)	7.6 (7.5–7.7) *	6.5 (6.4–6.6) *	5.2 (5.1–5.3) *	5.0 (4.9–5.1) *	4.7 (4.6–4.7) *
Pacemaker	5.3 (5.2–5.4)	5.4 (5.2–5.5)	4.7 (4.6–4.8) *	3.1 (3.0–3.2) *	3.2 (3.2–3.3) *	3.1 (3.0–3.2) *
Defibrillators	6.0 (5.8–6.3)	5.9 (5.6–6.1)	6.0 (5.8–6.3)	4.3 (4.1–4.5) *	4.2 (4.0–4.4) *	3.7 (3.5–3.9) *
ILR	14.1 (13.7–14.4)	14.0 (13.7–14.3)	10.4 (10.2–10.7) *	10.2 (10.0–10.5) *	8.9 (8.7–9.1) *	8.5 (8.3–8.7) *

*: *p* < 0.05 vs. 2018; #: event rates (95% confidence intervals).

## Data Availability

Data are contained within the article.

## References

[1] Crossley G.H., Boyle A., Vitense H., Chang Y., Mead R.H. (2011). The CONNECT (Clinical Evaluation of Remote Notification to Reduce Time to Clinical Decision) trial: The value of wireless remote monitoring with automatic clinician alerts. J. Am. Coll. Cardiol..

[2] Varma N., Michalski J., Epstein A.E., Schweikert R. (2010). Automatic remote monitoring of implantable cardioverter-defibrillator lead and generator performance: The Lumos-T Safely RedUceS RouTine Office Device Follow-Up (TRUST) trial. Circ. Arrhythm. Electrophysiol..

[3] Saxon L.A., Hayes D.L., Gilliam F.R., Heidenreich P.A., Day J., Seth M., Meyer T.E., Jones P.W., Boehmer J.P. (2010). Long-term outcome after ICD and CRT implantation and influence of remote device follow-up: The ALTITUDE survival study. Circulation.

[4] Hindricks G., Taborsky M., Glikson M., Heinrich U., Schumacher B., Katz A., Brachmann J., Lewalter T., Goette A., Block M. (2014). Implant-based multiparameter telemonitoring of patients with heart failure (IN-TIME): A randomised controlled trial. Lancet.

[5] Boriani G., Da Costa A., Quesada A., Ricci R.P., Favale S., Boscolo G., Clementy N., Amori V., Mangoni di S., Stefano L. (2017). Effects of remote monitoring on clinical outcomes and use of healthcare resources in heart failure patients with biventricular defibrillators: Results of the MORE-CARE multicentre randomized controlled trial. Eur. J. Heart Fail..

[6] Landolina M., Perego G.B., Lunati M., Curnis A., Guenzati G., Vicentini A., Marzegalli M. (2012). Remote monitoring reduces healthcare use and improves quality of care in heart failure patients with implantable defibrillators: The evolution of management strategies of heart failure patients with implantable defibrillators (EVOLVO) study. Circulation.

[7] Guédon-Moreau L., Lacroix D., Sadoul N., Clémenty J., Kouakam C., Hermida J.-S., Aliot E., Boursier M., Bizeau O., Kacet S. (2013). A randomized study of remote follow-up of implantable cardioverter defibrillators: Safety and efficacy report of the ECOST trial. Eur. Heart J..

[8] Tedeschi A., Palazzini M., Trimarchi G., Conti N., Di Spigno F., Gentile P., D’Angelo L., Garascia A., Ammirati E., Morici N. (2024). Heart Failure Management through Telehealth: Expanding Care and Connecting Hearts. J. Clin. Med..

[9] Ferrick A.M., Raj S.R., Deneke T., Kojodjojo P., Lopez-Cabanillas N., Abe H., Boveda S., Chew D.S., Choi J.I., Dagres N. (2023). 2023 HRS/EHRA/APHRS/LAHRS Expert Consensus Statement on Practical Management of the Remote Device Clinic. Europace.

[10] Maines M., Palmisano P., Del Greco M., Melissano D., De Bonis S., Baccillieri S., Zanotto G., D’Onofrio A., Ricci R.P., De Ponti R. (2021). Impact of COVID-19 Pandemic on Remote Monitoring of Cardiac Implantable Electronic Devices in Italy: Results of a Survey Promoted by AIAC (Italian Association of Arrhythmology and Cardiac Pacing). J. Clin. Med..

[11] Zanotto G., Melissano D., Baccillieri S., Campana A., Caravati F., Maines M., Platania F., Zuccaro L., Landolina M., Berisso M.Z. (2020). Intrahospital organizational model of remote monitoring data sharing, for a global management of patients with cardiac implantable electronic devices: A document of the Italian Association of Arrhythmology and Cardiac Pacing. J. Cardiovasc. Med..

[12] Maines M., Tomasi G., Moggio P., Peruzza F., Catanzariti D., Angheben C., Simoncelli M., Degiampietro M., Piffer L., Valsecchi S. (2020). Implementation of remote follow-up of cardiac implantable electronic devices in clinical practice: Organizational implications and resource consumption. J. Cardiovasc. Med..

[13] Maines M., Degiampietro M., Tomasi G., Poian L., Cont N., Peruzza F., Moggio P., Triglione F., Giacopelli D., Del Greco M. (2023). Strategic reprogramming of implantable cardiac monitors reduces the false-positive remote alert burden in a nurse-led service. Eur. J. Cardiovasc. Nurs..

[14] Maines M., Tomasi G., Moggio P., Poian L., Peruzza F., Catanzariti D., Angheben C., Cont N., Valsecchi S., Del Greco M. (2021). Scheduled versus alert transmissions for remote follow-up of cardiac implantable electronic devices: Clinical relevance and resource consumption. Int. J. Cardiol..

[15] Varma N., Love C.J., Michalski J., Epstein A.E., TRUST Investigators (2021). Alert-Based ICD Follow-Up: A Model of Digitally Driven Remote Patient Monitoring. JACC Clin. Electrophysiol..

